# Ion Channels in the Paraventricular Hypothalamic Nucleus (PVN); Emerging Diversity and Functional Roles

**DOI:** 10.3389/fphys.2018.00760

**Published:** 2018-07-06

**Authors:** Claire H. Feetham, Fiona O’Brien, Richard Barrett-Jolley

**Affiliations:** ^1^Faculty of Biology, Medicine and Health, University of Manchester, Manchester, United Kingdom; ^2^Institute of Ageing and Chronic Disease, University of Liverpool, Liverpool, United Kingdom

**Keywords:** PVN, paraventricular nucleus, hypothalamus, ion channels, hypertension

## Abstract

The paraventricular nucleus of the hypothalamus (PVN) is critical for the regulation of homeostatic function. Although also important for endocrine regulation, it has been referred to as the “autonomic master controller.” The emerging consensus is that the PVN is a multifunctional nucleus, with autonomic roles including (but not limited to) coordination of cardiovascular, thermoregulatory, metabolic, circadian and stress responses. However, the cellular mechanisms underlying these multifunctional roles remain poorly understood. Neurones from the PVN project to and can alter the function of sympathetic control regions in the medulla and spinal cord. Dysfunction of sympathetic pre-autonomic neurones (typically hyperactivity) is linked to several diseases including hypertension and heart failure and targeting this region with specific pharmacological or biological agents is a promising area of medical research. However, to facilitate future medical exploitation of the PVN, more detailed models of its neuronal control are required; populated by a greater compliment of constituent ion channels. Whilst the cytoarchitecture, projections and neurotransmitters present in the PVN are reasonably well documented, there have been fewer studies on the expression and interplay of ion channels. In this review we bring together an up to date analysis of PVN ion channel studies and discuss how these channels may interact to control, in particular, the activity of the sympathetic system.

## Introduction

### Location and Cytoarchitecture

The paraventricular nucleus of the hypothalamus (PVN) is one of the most important autonomic control centers in the brain with roles in a number of homeostatic responses (Badoer, 2001; Coote, 2007; Pyner, 2009; Nunn et al., 2011). In the rat, the PVN extends rostrocaudally below the third ventricle and is the most dorsal component of the thalamic midline-intralaminar nuclear complex (Paxinos and Watson, 1986). In both rodents and primates, neurones in the PVN connect extensively with a variety of neurones in the hypothalamus, brainstem, limbic regions and prefrontal cortex (Swanson et al., 1980; Swanson and Sawchenko, 1983).

**FIGURE 1:**
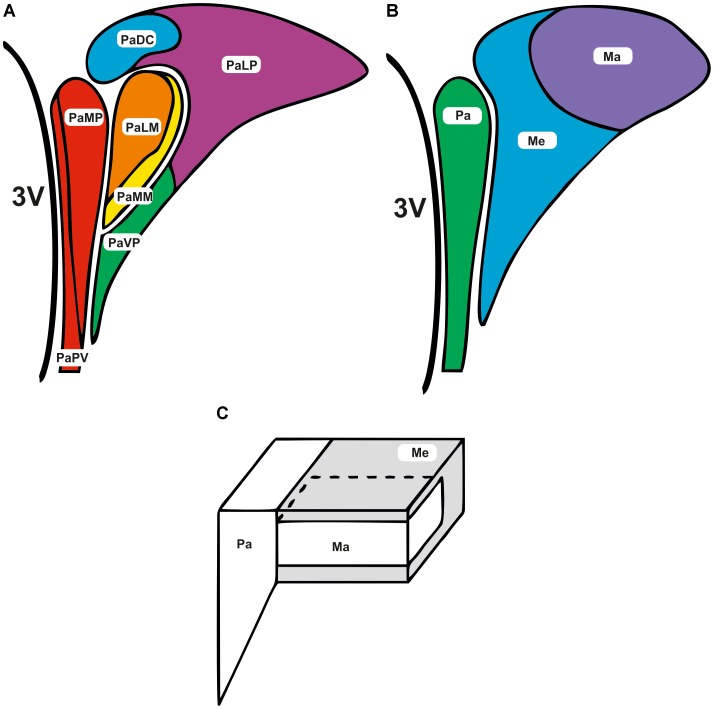
Subdivisions of the paraventricular nucleus. The subdivisions of the PVN are adjacent to the third ventricle (3V). **(A)** Parvocellular and magnocellular regions are further subdivided into the following areas; lateral magnocellular (PaLM), medial magnocellular (PaMM), medial parvocellular (PaMP), lateral parvocellular (PaLP), periventricular subnucleus (PaPV), medial ventral parvocellular (PaVP), and dorsal cap (PaDC) (Paxinos and Watson, 1986; Koutcherov et al., 2000).**(B,C)** Alternatively the PVN can be divided more loosely into the parvocellular (Pa), posterior magnocellular lateral (Ma), and the intermediocellular regions (Me) (Kiss et al., 1991; Chen et al., 2014).

The PVN comprises of several anatomical subdivisions, with the region typically divided into parvocellular and magnocellular subnuclei. The rat parvocellular area includes approximately 1000 small neurones that project to regions in the central nervous system (CNS) involved in autonomic control (Swanson and Kuypers, 1980; Lovick et al., 1993; Shafton et al., 1998; Pyner and Coote, 2000) via projections to the spinal cord (termed spinally projecting neurones) and medulla (Swanson and Kuypers, 1980; Hosoya et al., 1991; Shafton et al., 1998; Pyner and Coote, 2000). The magnocellular area contains larger neurones that project to the posterior pituitary. Cells within this area typically have a neuroendocrine function, for example, secretion of vasopressin and oxytocin. To date, 30 or more neurotransmitters have been identified in this region (Pyner, 2009). The parvocellular and magnocellular regions can be further subdivided (**Figure 1A**); although an alternative approach to anatomical subdivisions can be seen in **Figures 1B,C**. Here, the PVN is divided more loosely into the parvocellular area, posterior magnocellular lateral area and the intermediocellular region (dorsal and caudal PVN) where pre-autonomic neurones are most abundant (Kiss et al., 1991; Chen et al., 2014).

Much of the earliest anatomical information on these subnuclei originated from detailed studies in rats (Swanson et al., 1980; Swanson and Sawchenko, 1983; Simmons and Swanson, 2008). However, the mouse PVN has been described as an equally complex in structure, but with noticeable differences. Unlike the rat PVN (Swanson and Kuypers, 1980), the mouse PVN is not as well differentiated and magnocellular and parvocellular neurones are often indistinguishable (Biag et al., 2012). In contrast to numerous studies on the rat PVN, few studies have compared neuropeptide distribution in the mouse PVN (Castel and Morris, 1988; Shimada and Ishikawa, 1989; Broberger et al., 1999; Kadar et al., 2010).

### Functional Roles of the PVN

The broad range of PVN roles in physiological integration have been reviewed extensively (Swanson and Sawchenko, 1980; Baertschi et al., 1983; Ferguson et al., 2008), including influence over the sympathetic nervous system and have been implicated in the control of cardiovascular function for example, regulating the cardiac sympathetic afferent reflex (Zhong et al., 2008), regulation of blood volume (Lovick et al., 1993; Pyner and Coote, 2000), circadian regulation of blood pressure (Cui et al., 2001) and cardiovascular responses to stress (Jansen et al., 1995). Pharmacological or electrical stimulation of the PVN results in rapid rises in heart rate, blood pressure and renal sympathetic nerve activity (Martin et al., 1991; Kannan et al., 1989; Martin and Haywood, 1993; Duan et al., 1997; Zhang et al., 1997; Schlenker et al., 2001; Kawabe et al., 2009). There has been some controversy regarding whether pre-autonomic PVN neurones really are involved in the cardiovascular response to stress; with Dampney and others (Dampney, 1994; DiMicco et al., 1995; Fontes et al., 2001; DiMicco et al., 2002) ascribing this to a mis-classification of dorsomedial hypothalamic action, but as we discussed previously (Nunn et al., 2011), the consensus does seem that these neurones are “multi-functional.” The physiological role of the PVN extends further than control of cardiovascular function; the PVN has multiple diverse roles such as hormonal stress responses, circadian rhythm and thermoregulation (see Nunn et al., 2011 for a review). In addition to altering changes in blood pressure, spinally projecting PVN neurones are thought to play a role in blood volume regulation (Lovick and Coote, 1988, 1989; Lovick et al., 1993). For example, stimulation of atrial stretch receptors resulted in inhibition of renal sympathetic nerve activity and increased c-fos expression in the parvocellular PVN (Pyner et al., 2002). Furthermore, targeted injections at different sites within the PVN, mainly located in the dorsal parvocellular region, led to an increase in renal sympathetic nerve activity, which was reversed by reducing the injection volume (Deering and Coote, 2000). In addition, the PVN is thought to be involved in the control of energy balance through its influences on feeding, pituitary hormone secretion and the autonomic nervous system. In rats, stimulation of the PVN resulted in an increase in interscapular brown adipose tissue (BAT) temperature (Amir, 1990). This response could be inhibited by the sympathetic ganglionic blocker chlorisondamine, chloride or the β-adrenergic receptor antagonist, propranolol but not by hypophysectomy, indicating the involvement of the sympathetic nervous system. In a later study, injection of the gut hormone cholecystokinin (CCK), known to reduce food intake, into the third ventricle resulted in an increase in firing rate of sympathetic nerves to BAT (Yoshimatsu et al., 1993). A more recent study has illustrated that orexigenic neuropeptide Y (NPY)-containing neurones of the arcuate nucleus (Arc) control BAT thermogenesis and sympathetic outflow via tyrosine hydroxylase (TH) PVN neurones (Shi et al., 2013). Additional studies involving lesioning or injecting various peptides into the PVN have resulted in overeating/obesity or reduced eating/anorexia, suggesting the PVN is also involved in food intake (Leibowitz et al., 2007).

The PVN has important regulatory functions for hepatic glucose control, a subject that has been elegantly reviewed by O’Hare and Zsombok (2016). Retrograde neuronal tracing studies show that PVN projections directly innervate the liver through both the sympathetic and parasympathetic systems (la Fleur et al., 2000; Buijs et al., 2003; Kalsbeek et al., 2004). In several studies, some liver-related PVN neurones have been shown to express oxytocin and corticotrophin-releasing hormone, but not vasopressin (Buijs et al., 2003; Stanley et al., 2010).

The role of the PVN in the stress response has been well documented. Duan et al. (1997) showed that cardiovascular responses to stress can be mimicked by electrical stimulation of the PVN (Duan et al., 1997). In addition, parvocellular preautonomic neurones expressing tachykinin receptors have been implicated in having a role in the stress response (Herman and Cullinan, 1997) and work from our group has shown that the tachykinin, substance P (SP), activates spinally projecting neurones during electrophysiological recordings (Womack and Barrett-Jolley, 2007; Womack et al., 2007). The PVN is the main driver of hypothalamic-pituitary adrenal (HPA) responses and is controlled by peptidergic neuroendocrine neurones located in the medial parvocellular division (Herman and Cullinan, 1997; Myers et al., 2012; Flak et al., 2014). Chronic stress has been shown to induce neuronal plasticity by methods including activation of glutamatergic innervations of parvocellular PVN CRH neurones (Flak et al., 2009).

The suprachiasmatic nucleus of the hypothalamus (SCN) is the primary circadian pacemaker in mammals and sends both inhibitory and excitatory projections throughout the PVN (Hermes et al., 1996a,b; Kalsbeek et al., 2000; Cui et al., 2001). Bilateral injections of bicuculline into the PVN prevents light-induced inhibition of melatonin; this study concluded that the GABAergic projections from the SCN were responsible for regulating melatonin release (Kalsbeek et al., 2000). Furthermore, lesioning the PVN results in a lower night-time level of melatonin (Kalsbeek et al., 2000).

The PVN plays a role in osmoregulation. For historical context, the earliest electrophysiology on hypothalamic osmosensing showed that SON neurones are excited by increases in tonicity and hyperpolarized and inhibited by hypotonicity (Mason, 1980). Following this, Yamashita et al. (1984) included a temperature and osmolality study in an extensive PVN neuropharmacological program (Yamashita et al., 1984), proposing that only about 10% PVN neurones were inhibited by osmolality (i.e., excited by hypertonicity) with about 35% behaving in the opposite way. There are emerging links between hypothalamic osmolality and sympathetic activity in addition to the established hypothalamic-neurohypophesis pathway. For example, hypertonic saline injected into the hypothalamus yields a pressor response (Chen and Toney, 2001; Bourque, 2008; Chu et al., 2010) and hypotonicity decreases sympathetic nerve activity, blood pressure and heart rate (Bourque and Oliet, 1997). Toney and co-workers propose that these mechansisms serve to support blood pressure and heart rate in the face of dehydration (Holbein et al., 2014).

The PVN also has roles in thermoregulation; Inenaga et al. (1987) were the first group to show the presence of thermosensitive neurones in the PVN and there have since been several studies demonstrating a role for the PVN in thermoregulation and thermogenesis (Cham et al., 2006; Cham and Badoer, 2008; Chen et al., 2008; Leite et al., 2012). Poly-synaptic tracing using the pseudorabies virus in rats identified spinally projecting neurones of the PVN specifically as being directly involved in thermoregulation (Leite et al., 2012).

### Electrophysiological Phenotypes of PVN Neurones

The electrophysiological properties of neurones within the PVN have been characterized in several studies. Early work used patch-clamp electrophysiology to record the electrical properties of hypothalamic neurones and labeled cells by injecting Lucifer yellow, ethidium bromide or biocytin (Hoffman et al., 1991). Further work by this group and others described three distinct types of PVN neurones based on their electrophysiological properties and these classifications are still useful today (see for example Lee et al., 2012). Type I neurones were identified as neurosecretory magnocellular neurones with phasic bursting patterns, expressing a rapidly inactivated, or “A-type,” potassium conductance (Tasker and Dudek, 1993; Sonner and Stern, 2005). Type II (parvocellular) neurones express a slowly inactivating delayed rectifier potassium conductance. The differences between types I and II cells may be explained by differential expression of voltage-gated potassium and calcium channels (Luther and Tasker, 2000). Furthermore, Tasker and Dudek (1991) described two different neuronal phenotypes within the parvocellular area; (1) exhibiting electrophysiological properties similar to neuroendocrine magnocellular cells and (2) pre-autonomic neurones (Tasker and Dudek, 1991). In the parvocellular subnuclei, medulla-projecting neurones show strong inward rectification and “A-type” potassium conductance (Stern, 2001; Sonner and Stern, 2005) whereas spinally projecting neurones show a slowly inactivating potassium conductance (Barrett-Jolley et al., 2000).

Ion channel expression is the key determinant of neuronal function; to model, understand and exploit the therapeutic potential of the PVN it will be necessary to establish which ion channels are present in the first place. Sonner et al. (2011) have already shown that disruption of ion channel regulation at the level of the PVN can lead to increased neuronal excitability of pre-sympathetic neurones, potentially contributing to sympathetic over activity (Sonner et al., 2011). Furthermore, the remarkable recent work of Geraldes et al. (2014, 2016, 2018) (discussed later) highlight the rich diversity of ways in which this could be exploited in the future.

### Ion Channels of the PVN

An ion channel is a pore-forming protein found on a cell membrane that permits the transport of ions, for example potassium, sodium and/or calcium. The main roles of ion channels include, but are not limited to, setting the resting membrane potential, shaping the action potential, transmitting electrical signals and acting as biological sensors (Clapham, 2003). As discussed above, the PVN has multiple, diverse roles resulting from neurone-to-neurone communication and propagation of electrical impulses from other areas of the brain. Excitability of PVN neurones results from the properties and distribution of ion channels on the plasma membrane, therefore, it is likely that any modification in ion channel function/distribution may affect the integration and propagation of such electrical signals.

## Amino Acid Receptor Channels

**FIGURE 2:**
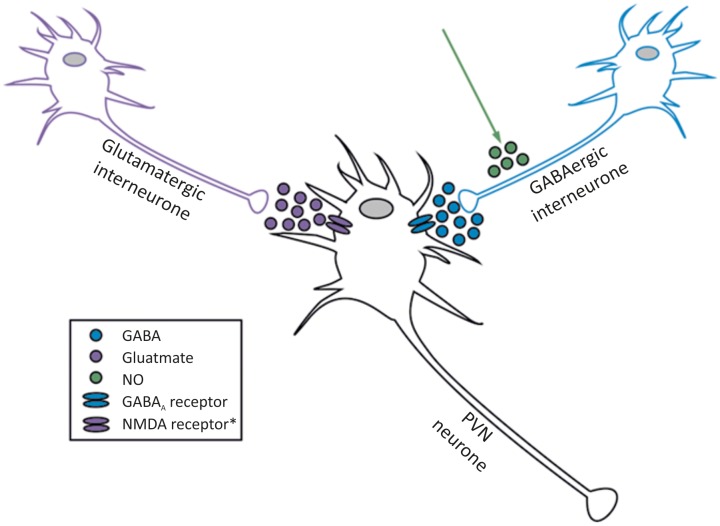
Interaction between glutamate, GABA and NO acting on neurones within the PVN. The two best established inputs to PVN spinally projecting neurones are GABA (inhibitory) and glutamate (excitatory), but NO is one of several factors that amplifies the GABA input. Spinally projecting sympathetic neurones are influenced by NO, but do not produce it themselves (Watkins et al., 2009). ^∗^NMDA, kainate and AMPA receptors have been identified in the PVN, please see full text.

Probably the earliest identified and best studied ion channels in the PVN are GABA and glutamate receptors (**Table 1**). This is unsurprising as GABA and glutamate are the most widely distributed inhibitory and excitatory neurotransmitters in the brain, respectively. Inputs to the PVN arise from the “time-keeper” SCN via glutaminergic input (Cui et al., 2001). The PVN is kept tonically inactive through the activity of surrounding GABA neurones (Martin and Haywood, 1992, 1993). Indirect inhibition of GABA is one mechanism by which several agents modulate pre-sympathetic neurones; nitric oxide (NO) (Li et al., 2002, 2004a), angiotensin (Li et al., 2003) and substance P (SP) act on GABA neurones in this way (**Figure 2**) (Womack et al., 2007).

**Table 1:** Amino acid receptor channels in the PVN.

Ion channel family	General role	Subtype identified	Method	Relevance	Reference
GABA_A_	Inhibition of neuronal activity/iPSP	Type A (subtype not specified)	Implicit from *in vivo* study.	Keeps spinally projecting neurones tonically inhibited	Martin et al., 1991; Martin and Haywood, 1993
			Patch-clamp electrophysiology		Li et al., 2002, 2004b; Zaki and Barrett-Jolley, 2002; Womack et al., 2006
		GABA_A_ α2-subunit	Immunohistochemistry	Also seen specifically in spinally projecting neurones	Fritschy and Mohler, 1995; Nunn et al., 2011
		α5-subunit	Immunohistochemistry	Decreased in hypertension	Cullinan, 2000
		α2, β1, and β3 subunits	qPCR	Especially in CRH positive neurones	Wang et al., 2009
GABA_B_	Inhibition of neuronal activity	–	Patch-clamp electrophysiology (post synaptic potentials).	Further inhibition of spinally-projecting neurones	Chen and Pan, 2006; Wang et al., 2009
Glutamate	Excitation/ePSPs	NMDA, AMPA	Patch-clamp	Activation of spinally-projecting neurones	Cui et al., 2001
	Excitation/ePSPs	GluR5; Kainate (KAR)	*In situ* hybridization and immunohistochemical plus a cFos study/pharmacology		Evanson et al., 2009


GABA_A_ receptor whole-cell (Womack et al., 2006) currents, single channel activity (Barrett-Jolley, 2001) and gene expression have all been characterized throughout the PVN in a number of studies. For example, Cullinan (2000) detected mRNA of α2, β1, and β3 subunits in >94% of CRH neurons and α1 and β3 subunits detected in >53% and >65% of CRH neurones, respectively. In addition, mRNA levels of the GABAα_1_ receptor types were shown in the PVN (Wang et al., 2009). Wang et al. (2009) also explored functional roles of GABA receptors in the PVN; injection of the GABAα receptor agonist muscimol or the GABA_β_ receptor agonist baclofen into the PVN resulted in similar, dose-dependent reductions in heart rate, mean arterial pressure and renal sympathetic activity (Wang et al., 2009).

*In situ* hybridization and immunohistochemical analyses confirmed the existence of the GluR5 kainate subunit in the PVN. Intra-PVN infusion of the kainate antagonist LY382884 increased plasma adrenocorticotropin (ACTH), corticosterone and PVN *c-fos* immunoreactivity (Evanson et al., 2009). We discuss the “energy sensing” K_ATP_ channel later, but PVN GABA-neurotransmission is also critical for metabolic regulation, where alterations at the level of the PVN contribute to dysregulation of hepatic functions including glucose metabolism. Administration of the glutamate agonist N-methyl-d-aspartate (NMDA) and the GABA_A_ antagonist bicuculline into the PVN significantly increased plasma glucose and glucagon levels, without change in plasma insulin. Interestingly, this response was absent in rats following sympathectomy (Kalsbeek et al., 2004).

## Transient Receptor Potential Channels

The parvocellular PVN, above everything else, is an integration center, receiving inputs from a number of sources to modulate sympathetic output. These include synaptic inputs from the SCN, an area of the hypothalamus controlling circadian rhythm and the cardiovascular autonomic control centers (Nunn et al., 2011). The PVN also plays a role in sensing changes in temperature, glucose, osmolality and other homeostatic processes (Cham and Badoer, 2008; Melnick et al., 2011; Feetham and Barrett-Jolley, 2014; Feetham et al., 2015b). Therefore, it is likely that this region is richly populated with ion channels which we hypothesize act as biological sensors (Clapham, 2003), such as the transient receptor potential (TRP) ion channels. The TRP multigene superfamily encodes a family of 28 ion channels that are conserved in yeast, invertebrates and vertebrates. The TRP family is divided into seven subfamilies, TRPC (canonical; seven members), TRPV (vanilloid; six members), TRPA (ankyrin; 1 member), TRPM (melastatin; eight members), TRPML (mucolipin; 1 member), TRPP (polycystin; two members) and TRPN (NOMPC; only seen so far in non-mammals) (Li et al., 2006). The majority of TRPs are termed non-selective cation channels, although most have greater selectivity for Ca^2+^ ions than monovalent cations. The TRP family has multiple biological roles as key molecular sensors both inside and outside the CNS and channel activation can vary from ligand binding to voltage, chemical, physical or thermal challenge. Since TRP channels usually have equal selectivity for both Na^+^ and K^+^ ions it would typically suggest that TRP channel activation would depolarize the cell membrane and activate voltage-dependent ion channels. However, both our experimental and modeling data show that TRP activation increases intracellular Ca^2+^ concentration and results in secondary activation of Ca^2+^-activated potassium channels (see TRPV4 below) and membrane hyperpolarization (Feetham et al., 2015b).

Several TRP channels have been identified in the PVN (**Table 2**). Interestingly, patch-clamp electrophysiology on parvocellular PVN neurones shows that angiotensin II, in addition to its modulation of GABA-ergic neurotransmission as discussed earlier, also results in cell depolarization through the modulation of non-selective cationic and potassium conductances in rats (Latchford and Ferguson, 2005). It is plausible that this non-selective cation conductance is mediated by TRP channels and that they can act as signals downstream of receptor activation in the PVN. In another study, leptin was found to increase a non-selective cation conductance and depolarize both type I and II PVN neurones, suggesting that neurones in the PVN are involved in the physiological response to leptin via non-selective cationconductances (Powis et al., 1998). Although the identity of the specific ion channels responsible is still unknown, there is evidence to suggest that TRP channels may be involved. For example, a study investigating the effect of leptin on PVN mediated sympathetic output (Zheng et al., 2014) found that sympathetic activity was almost abolished by the TRPC inhibitors 2APB and SKF96365. However, it is worth noting that a caveat with any pharmacological inhibition of TRP channels is that the modulators are often *not* as selective as one would hope.

**Table 2:** TRP channels in the PVN.

Ion channel family	General role	Subtype identified	Method	Relevance	Reference
TRPC	Calcium homeostasis, open with ↓ Ca^2+^	TRPC4	RT-PCR, western blot	Expression increased following water deprivation ↑	Hindmarch et al., 2006; Fowler et al., 2007; Zheng et al., 2014
Canonical		TRPC1-6	RT-PCR Patch-clamp electrophysiology	Expression contributes to inappropriate vasopressin release in cirrhosis.	
Store-operated		TRPC			
TRPV	Mechanosensitive and homeostatic roles	TRPV1	Radiolabeling, patch clamp electrophysiology Immunohistochemistry, Western blot, patch clamp electrophysiology, *in vivo* studies	Activation leads to glutamate release and postsynaptic firing. Co-localized with liver-related neurones Regulates body fluid homeostasis, autonomic control and metabolism Pharmacological activation of TRPV4 ↓ firing in brain slice, depolarization and Ca^2+^ rise in isolated neurones. TRPV4 inhibitors reverse hypotonic effects on firing.	Li et al., 2004b; Roberts et al., 2004; Carreno et al., 2009; Zsombok et al., 2011; Gao et al., 2012; Nedungadi et al., 2012; van den Burg et al., 2015; Feetham et al., 2015a
Vanilloid		TRPV2			
		TRPV4			
TRPM Melastatin	Sensor/homeostatic roles, exhibit temperature sensing properties	TRPM4 and TRPM5	Confocal immunofluorescence	Differential expression dependent upon area of neurone	Teruyama et al., 2011


### TRPC (Canonical Transient Receptor Potential) Channels

The TRPCs are extensively expressed in the brain (Li et al., 1999) but also expressed in other organs for example, the heart, kidneys and the lungs. Of the seven TRPC channel members identified, TRPC4 is the predominant subtype in the rat brain (Fowler et al., 2007). TRPCs have been identified in the PVN using a variety of techniques; for example, TRPC4 and TRPC5 mRNA were detected using *in situ* hybridization (Fowler et al., 2007). In addition, microarray analysis also confirmed the presence of TRPC4 in the PVN (Hindmarch et al., 2006). This study also aimed to investigate the role of TRPC4 in the PVN with TRPC4 expression increased following water deprivation (Hindmarch et al., 2006). This “transcriptomic” study suggests that TRPC4 may be differentially expressed as a part of an adaptive response to hyper-osmolality. However, the size of the effect, specifically in the PVN, is unclear since more recent qPCR data shows a large increase in SON TRPC4 mRNA in water deprivation (whole tissue homogenates), but little change in the PVN itself (Nedungadi and Cunningham, 2014). We subsequently have shown that TRPV4 has a central role in osmoregulation in the PVN using patch clamp electrophysiology, however, it is likely that other channels, such as TRPC channels may also be involved (Feetham et al., 2015b).

Osmoregulation is not the only role for TRPCs in the PVN; they also appear to contribute to the elevated sympathetic activity seen in type 2 diabetes (T2D) (Zheng et al., 2014). PVN qPCR data show increased mRNA expression of several subtypes of TRPC channel (TRPC1, 4, 5, and 6) following streptozotocin induced T2D (see “PVN Ion Channels in Disease” section below). Parallel *in vitro* data suggest that this phenomenon results from the action of the energy regulating hormone leptin (Zheng et al., 2014) that is increased in diabetes (Pan et al., 2014).

### TRPV (Vanilloid Transient Receptor Potential) Channels

In the periphery, TRPV channels are associated with pain, mechanosensation (Guilak et al., 2010) and thermoregulation (Caterina, 2007). The majority of studies on the functional/structural properties of TRPV channels in the CNS have focused on TRPV1. However, at least 5 other TRPV channels have been identified in mammals including TRPV2, TRPV3, and TRPV4, all of which are found in the CNS (Caterina, 2007). TRPV channels have been widely reported in the PVN; to summarize, TRPV1, TRPV2, TRPV4, and TRPV5 channel expression has been reported. A thorough autoradiography study using TRPV1 null mice as a negative control identified weak TRPV1 protein expression in the PVN of mice (Roberts et al., 2004). This is backed up by patch clamp brain slice data in rats, showing TRPV1 channel dependent activation of glutaminergic neurotransmission (Li et al., 2004b). Additionally, combined viral retrograde tracer and immunohistochemical data show TRPV1 expression in liver projecting pre-autonomic PVN neurones, and co-localization with insulin receptor 2 (Zsombok et al., 2011; Gao et al., 2012); suggesting that TRPV1 in the PVN appears to play a role in the control of hepatic glucose production. The hypothesis is that this PVN projection serves to couple liver glucose production with other physiological and endocrine PVN targets via increases of (liver) sympathetic activity. This, along with the TRPC data discussed above, demonstrates the striking potential of ion channel modulation at the level of the PVN to ameliorate sympathetic activity in a range of cardiovascular and metabolic disorders.

Immunohistochemical techniques in the rat brain have also identified TRPV2 in the magnocellular region of the PVN with some staining also observed in the posterior parvocellular region and dorsal horn (Nedungadi et al., 2012). This study also revealed co-localization of TRPV2 with vasopressin-expressing cells in the magnocellular PVN, further highlighting the complexity of TRP channel mediated osmoregulation in the PVN. A further role for TRPV2 has also been proposed in primary hypothalamic neurones; where oxytocin, one of the many PVN neuropeptide transmitters, has been shown to induce Ca^2+^ dependent signaling via activation of TRPV2 channels. One immunohistochemistry study revealed co-localization of TRPV2 and oxytocin in the PVN, suggesting that TRPV2 may be a mediator of the anxiolytic effects of oxytocin (van den Burg et al., 2015). Since oxytocin appears to have other roles too, it will be interesting to discover if they too are mediated by TRPV2. It is quite likely that in native tissue, combinations of different ion channels are involved with any given response and this is emphasized by similar data showing TRPV5 mediated oxytocin (and vasopressin) responses (Kumar et al., 2017). This TRPV5 expression study used immunohistochemistry and target verification with siRNA knock-down of the channel (Kumar et al., 2017). TRPV5 has been observed in both neurones and glia, although the role of TRPV5 in glia is yet to be established. This study showed TRPV5 expression in a remarkable ∼50% of vasopressin neurones and ∼6–7% of oxytocin neurones in the rat PVN (Kumar et al., 2017). It is therefore likely that TRPV2 and TRPV5 work in a coordinated fashion in the maintenance of endocrine homeostasis.

**FIGURE 3:**
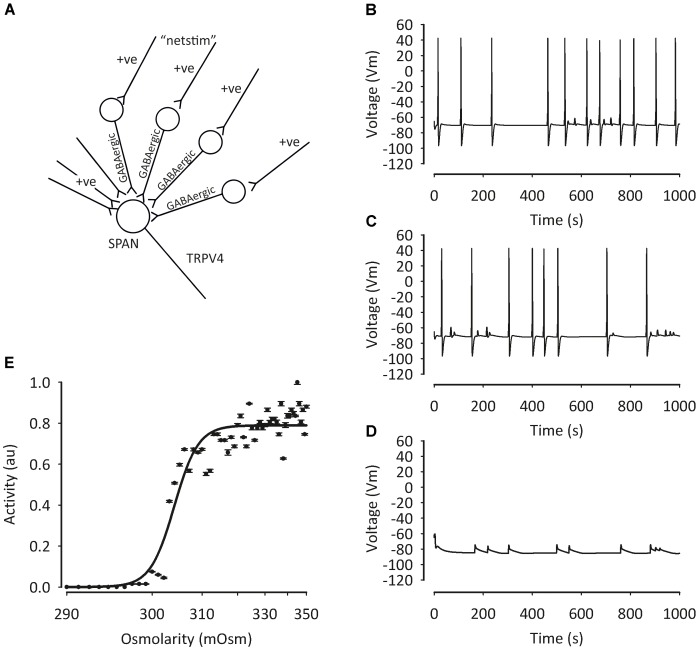
Mathematical model of pre-autonomic neurone regulation by osmolality. To test the plausibility of our PVN osmolality conceptual model, we deployed a mathematical model written in NEURON (Feetham et al., 2015b), including intracellular Ca^2+^ buffering. TRP channels were constructed as relatively non-specific cation channels with ratio (Ca^2+^: K^+^: Na^+^) 6:1:1 and activated by hypotonicity. **(A)** Sympathetic pre-autonomic neurones (SPANS) receive excitatory and inhibitory input. Despite the activation of a non-specific cation channel conductance action potential frequency decreased **(B)** 320 mOsm, **(C)** 300 mOsm **(D)** 280 mOsm. Data summarized in **(E)**. Figure from Feetham et al. (2015b) with permission of the copyright holder John Wiley & Sons, Inc.

**FIGURE 4:**
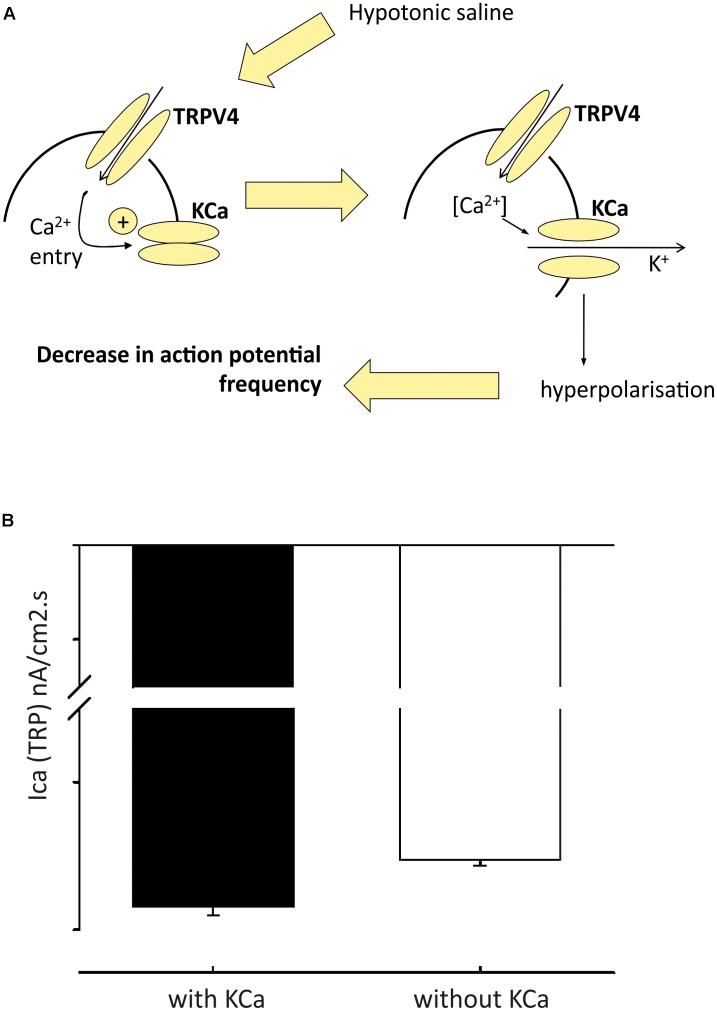
Model of pre-autonomic neurone regulation by osmolality. From detailed analysis of the model (in **Figure 3**) we predicted that inward TRP dependent Ca currents would be increased in the presence of K_Ca_ block **(A)**; and we then validated this by experiment **(B)** Figure from Feetham et al. (2015b) with permission of the copyright holder John Wiley & Sons, Inc.

Western blot studies have shown that TRPV4 is also expressed in the PVN (Carreno et al., 2009). Our own group has confirmed the presence of TRPV4 in the PVN using immunohistochemistry and patch-clamp electrophysiology. Our data show that TRPV4 is involved in osmosensing in the PVN (Feetham et al., 2015a,b). We investigated the detailed mechanism of hypotonicity mediated decreases in parvocellular PVN neurones with a series of *in vivo*, *in vitro* and *in silico* studies (Feetham et al., 2015a,b). We found TRPV4 to be central to the PVN hypotonic response, but the mechanism underlying these responses involved a rise in intracellular Ca^2+^, which could possibly be missed with whole-cell patch clamp recordings. Our evidence was briefly as follows; following addition of TRPV4 agonists, a decrease in firing rate was observed similar to that seen during hypotonic challenge in the absense of TRPV4 activation. In addition, we reported that the decrease in firing with hypotonic challenge could be reversed with TRPV4 specific inhibitors. In whole-cell experiments, addition of TRPV4 activators led to the depolarization of isolated PVN neurones from rats, but an increase in intracellular Ca^2+^ was observed in intact cells (Feetham et al., 2015a). We created (**Figure 3**) and validated (**Figure 4**) this model numerically using a mathematical model written in NEURON and whilst less mechanistic, we verified that TRPV4 was involved with central osmosensing at the whole animal level *in vivo* experiments; central injections of hypotonic solution decreased blood pressure and this effect was blocked by a TRPV4 inhibitor (Feetham et al., 2015a).

### TRPM (Melastatin Transient Receptor Potential) Channels

Of this family, the best characterized channels in the PVN are TRPM4 and M5 (Teruyama et al., 2011). These are closely related channels with 40% amino acid sequence homology and are rather distinct from most other TRP channels (including the other melastatin TRPs) in that they are Ca^2+^ *impermeant* (Liman, 2014). Both these channels are of relatively low conductance [in the order of 20pS (Nilius et al., 2003; Zhang et al., 2007)] and TRPM5 is a well-established chemical sensor. TRPM5 is best characterized for gut chemosensing and for sensing bitter, sweet and umami in gustatory neurones (Perez et al., 2002). It seems reasonable to speculate that TRPM4 and TRPM5 in the PVN may therefore serve as sensors, extending the integrative capacity of the PVN to chemical sensing. TRPM4 channels are sensitive to pH and reactive oxygen species and intracellular ATP levels (Mathar et al., 2014). It is therefore possible that they contribute to the changes in activity of the PVN seen with inflammaging (Zubcevic et al., 2011). However, thus far they have only been identified by confocal immunofluorescence in magnocellular regions (Teruyama et al., 2011), and not the parvocellular subnuclei directly associated with sympathetic control.

## Potassium Channels

K^+^ channels are the most widely distributed ion channel and are found in virtually all living organisms. They have a multitude of physiological functions making them common therapeutic targets (Humphries and Dart, 2015). Encoded by KCN genes, K^+^ channels form membrane spanning pores that are selective for K^+^ ions (Alexander et al., 2015). Many subfamilies exist and are named from the physiological signals by which the pore opening is affected, for example, voltage and Ca^2+^ -gated channels. Based on structure and function, K^+^ channels generally have three major classes: the voltage-gated K_v_ (including KCNA) with six transmembrane domains, inward rectifying, Kir (including KCNJ) with 2 transmembrane domains and the KCNK tandem pore domain (K2P) with 4 transmembrane domains.

### Voltage-Gated Potassium Channels

K_v_ channels open and close in response to changes in membrane potential, allowing passive flow of K^+^ from the cell to restore membrane potential. They are key players in propagating electrical impulses in nerves as originally quantified by Hodgkin and Huxley (1952). The K_v_ family includes some 40 voltage-gated potassium channel genes including KCNA which encodes the K_v_.1x channels (Coetzee et al., 1999). Several K_v_ channel subtypes have been identified in the PVN (**Table 3**), and the differences in electrophysiological properties of PVN neurones may be due to differences in K_v_ expression levels. For example, immunohistochemical evidence has suggested that the “A-type” potassium current from PVN-RVLM neurones is mediated by KCNA4 (K_v_1.4) and/or KCND3 (K_v_4.3) channel subunits (Sonner and Stern, 2007). In contrast, the K_v_ currents observed in identified spinally projecting neurones appeared to be delayed rectifier in nature with no “A-type” potassium current observed (Barrett-Jolley et al., 2000). Further subtypes of voltage-gated potassium channels have also been identified within rat PVN neurones. In a study using a combination of patch-clamp electrophysiology and single cell reverse-transcriptase polymerase chain reaction (RT-PCR) in PVN neurones in rats, expression profiles of KCNA2 (K_v_1.2), KCNA3 (K_v_1.3), KCNA4 (K_v_1.4), KCND1 (K_v_4.1), KCND2 (K_v_4.2), and KCND3 (K_v_4.3) in both types of neurones were identified, with some level of co-expression. KCND2 (K_v_4.2) and KCND3 (K_v_4.3) expression was shown to be higher in type I neurones, making it possible to distinguish between PVN cell types (Lee et al., 2012). The presence of KCNA2 (K_v_1.2) has also been identified within rat PVN neurones using immunohistochemistry, with researchers reporting an increase in PVN KCNA2 (K_v_1.2) expression following focal ischemic injury (Chung et al., 2001). Intuitively, increased expression of potassium channels may reduce firing rate and so may be a self-protective mechanism. In addition, KCNA2 (K_v_1.2) along with KCNA1 (K_v_1.1) channels have been shown to be downstream effectors of NO on synaptic GABA release to identified spinally projecting neurones of the rat PVN (Yang et al., 2007).

**Table 3:** Potassium channels in the PVN.

Ion channel family	General role	Subtype Identified	Method	Relevance	Reference
K_V_ Voltage-activated	Controlling action potential characteristics, modulate neuronal excitability	K_V_1.1 and K_V_1.2	Immunohistochemistry, patch clamp electrophysiology	Downstream effectors of NO on synaptic GABA release	Chung et al., 2001; Yang et al., 2007
		K_V_1.4 and K_V_4.3	Immunohistochemistry	“A”-type K^+^ current	Sonner and Stern, 2005; Sonner and Stern, 2007; Sonner et al., 2008
		K_V_1.2, K_V_1.3, K_V_1.4, K_V_4.1, K_V_4.2 and K_V_4.3	Patch clamp electrophysiology and RT-PCR	↑ expression of K_V_4.2 and K_V_4.3 in “type I” neurones	Lee et al., 2012
		K_V_7.2 and K_V_7.3	Patch clamp electrophysiology, ICV *in vivo* injections.	Acute stress decreases expression of K_V_7.3 and blunts M-current in CRH neurones.	Zhou et al., 2017
K_Ca_	Regulate neuronal excitability.	BK	Immunohistochemistry	Voltage-dependant	Sausbier et al., 2006
Calcium-activated	Contribute to after-hyperpolarization				
		SK1, SK2, and SK3	Immunohistochemistry, patch-clamp electrophysiology, targeted *in vivo* injections	Inhibition leads to ↑ excitability, ↑RSNA, ↑SSNA ↑HR, ↑BP Decreased functionality ↑hyperexcitability	Kohler et al., 1996; Chen and Toney, 2009; Gui et al., 2012; Pachuau et al., 2014
K_ATP_ ATP-sensitive	Modulate excitability	–	Patch-clamp electrophysiology, computational modeling	Adenosine ↓excitability via adenosine receptors	Lewis et al., 2010; Li et al., 2010
GIRK g-protein coupled inwardly rectifying	Synaptic inhibition through activation of GPCRs	GIRK1, GIRK2, and GIRK3	Immunohistochemistry, *in situ* hybridization	Role in presynaptic inhibition of neurotransmitter release?	Morishige et al., 1996; Shirasaka et al., 2007; Saenz del Burgo et al., 2008
Kir	Diverse functions such as maintaining action potential and regulating insulin release	Kir2.1		Overexpression of Kir2.1 controls excitability and influences sympathetic nervous system	Geraldes et al., 2014
K2P	Similar to inward rectifier, but pH sensitive	TASK-like	Patch-clamp	Conveys pH sensitivity	Doroshenko and Renaud, 2009


The M-current is a slowly developing, non-inactivating, time- and voltage-dependent potassium current that was initially described in bullfrog sympathetic ganglia (Jones, 1987). It helps maintain the resting membrane potential of neurones and is mediated by KCNQ2 (K_v_7.2) and KCNQ3 (K_v_7.3) subunits (Coetzee et al., 1999). The PVN is rich in SP and interestingly, one of the first known modulators of the M-current was SP (Stanfield et al., 1985; Stonehouse et al., 1999), although this mechanism has not yet been characterized in the PVN. However, in a recent study, KCNQ2 (K_v_7.2) and KCNQ3 (K_v_7.3) have been identified specifically in CRH-expressing neurones within the PVN (Zhou et al., 2017). In this study, acute stress decreased KCNQ3 (K_v_7.3) expression in the PVN of rats and blunted the excitatory effect of the KCNQ (K_v_7) channel blocker, XE-991, on firing activity. These data suggest that acute stress-caused hyperactivity of the HPA axis is due to a dysfunction of KCNQ (K_v_7) channels (Zhou et al., 2017), which in turn, results in increased AMPK activity.

### Calcium Activated K^+^ Channels

Calcium activated K^+^ channels are a large family of K^+^-selective channels with 6/7 transmembrane domains that are abundant throughout the CNS (Faber and Sah, 2003). They are activated by rises in cytosolic calcium, which may occur when Ca_v_ channels open during action potentials, leading to an influx of calcium. Three general K_Ca_ subfamilies have been identified, which can be distinguished based on biophysical and pharmacological differences: large-conductance KCNMA1 (BK), intermediate-conductance KCNN4 (IK/SK4) and small conductance KCNN1-3 channels (SK1, SK2 and SK3) (Sah, 1996; Vergara et al., 1998).

BK (encoded by KCNMA) and SK (encoded by one of four KCNN genes) channels are known to modulate firing in the brain (Kohler et al., 1996; Sausbier et al., 2006). Whilst one can confidently distinguish BK from SK channels pharmacologically, distinction of different KCNN subtypes is more difficult and so we use the generic term “SK” below. Patch-clamp electrophysiology recordings revealed the presence of an SK current in PVN-RVLM projecting neurones (**Table 3**). Inhibition of these channels with the SK channel blockers apamin and UCL1684, led to increased excitability of PVN-RVLM neurones in rats (Chen and Toney, 2009). *In vivo* investigations using targeted injections of pharmacological inhibitors of SK into the PVN induced increases in sympathetic nerve activity, heart rate and blood pressure (Gui et al., 2012). Although this work compliments the *in vitro* findings of Chen and Toney (2009), it is not possible to identify the neuronal population responsible for the responses observed. Recent evidence implicates a role for spinally projecting PVN neurones; patch-clamp recordings show that SK channel activity is reduced in hypertension (Pachuau et al., 2014), modulated by casein kinase II upregulation. This decreased functionality of SK contributes to the hyperactivity of these neurones (Pachuau et al., 2014). To add complexity to the role of SK, several investigations have suggested functional couplings to non-selective cation channels such as the transient receptor potential (TRP) channels (Gao and Wang, 2010; Earley, 2011). Work from our own group has verified the presence of SK channels in the PVN using immunohistochemistry and patch-clamp electrophysiology. We show that SK channels functionally couple to TRPV4 channels in the PVN (Feetham et al., 2015b). We discuss the mechanism above (including our mathematical model in **Figures 3**, **4**), but further details include the fact that the K_Ca_ channel involved in PVN osmosensing is SK and therefore sensitive to the antagonist UCL-1684.

Immunocytochemical staining of rat hypothalamic neurones has demonstrated BKβ1 (KCNMB1) expression in the PVN (Salzmann et al., 2010). In addition, a functional role for BK channels in the HPA-axis was investigated using genetically deficient mice for the pore forming KCNMA1 (BK) subunits (BK-/-). Reduced activation of hypothalamic PVN neurones was observed in response to stress in the BK-/- mouse, demonstrating an important role for KCNMA1 (BK) channels in HPA function in stress (Brunton et al., 2007).

### Inwardly Rectifying K^+^ Channels

Inwardly rectifying channels were first described in skeletal muscle, where a greater K^+^ current *into* rather than *out of* the cell was observed; because this behavior is counter intuitive, it is sometimes referred to as the anomalous rectifier (Kubo et al., 1993). Broadly speaking there are strong and weak inward rectifier channels; the strong rectifiers including Kir2.x (KCNJ2), generally prevent any outward current flow (Dart et al., 1998), whereas the weaker inward rectifiers including Kir6.2 (KCNJ11) merely show inward rectification (Wellman et al., 1999).

These characteristics are very different from the voltage-gated K^+^ currents discussed above (Nichols and Lopatin, 1997) as they have a tendency to electrically stabilize neurones near to the resting membrane potential, but inactivate once depolarization has been generated. Encoded by several KCNJ genes, they have diverse physiological functions such as regulating insulin release in pancreatic β-cells or modulation of heart rate myocytes (Nichols and Lopatin, 1997). There are seven subfamilies, but these are typically divided into 4 groups: Classical KCNJ2 channels (Kir2.x), G protein-gated KCNJ3 channels (Kir3.x), ATP-sensitive KCNJ11 channels (Kir6.x) and KCNJ transport channels (Kir1.x, Kir4.x, Kir5.x, and Kir7.x).

As previously mentioned, neurones in the PVN show an inwardly rectifying potassium current (**Table 3**). In studies by Geraldes et al. (2014, 2016) the inwardly rectifying potassium channel, Kir2.1 was overexpressed in spontaneously hypertensive rats using the lentivirus, leading to decreased blood pressure and decreased sympathetic output (Geraldes et al., 2014, 2016). G-protein coupled inwardly-rectifying potassium (GIRK) channels have also been shown to be widely distributed throughout the rodent brain. These channels are activated by several neurotransmitters and are important in synaptic inhibition (Brown, 1990; Hille, 1994), mediating the regulation of neuronal excitability via activation of G-protein coupled receptors. Three out of 4 GIRK subunits, GIRK1, GIRK2, and GIRK3 have all been shown to be present in the PVN using immunohistochemistry and *in situ* hybridization (Saenz del Burgo et al., 2008). GIRK1 specifically has been shown to be expressed presynaptically in the PVN, potentially having a role in presynaptic inhibition of neurotransmitter release (Morishige et al., 1996).

**FIGURE 5:**
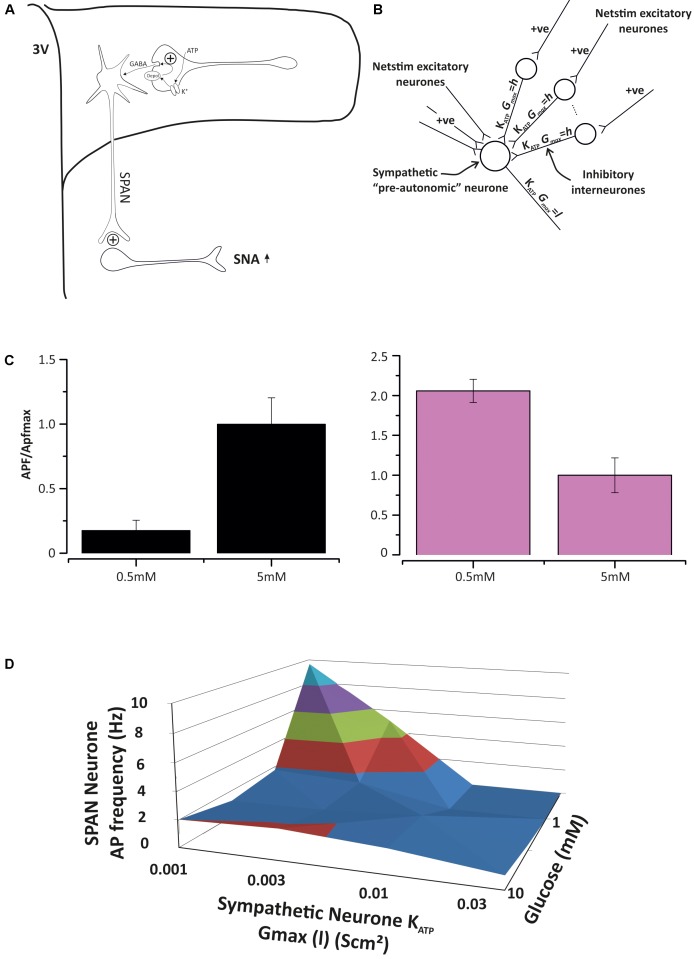
Model of pre-autonomic neurone regulation by K_ATP_ channels. **(A)** The base model, spinally-projecting neurones (SPAN) receive excitatory and inhibitory input in the PVN and activate spinal preganglionic neurones. Both the SPAN and inhibitory interneurons express K_ATP_ channels, but at different densities (Shiraishi, 1987). **(B)** Parameterizing the model with different K_ATP_ conductances (maximum) leave the cell to respond differently to glucose levels. **(C)** On the left, with low SPAN K_ATP_ expression, increasing glucose excites the SPAN, but when K_ATP_ expression is sufficiently high, elevated glucose will decrease action potential frequency (APf). This paradox is accounted for by the relatively greater effect of glucose on the inhibitory input neurone. **(D)** A 3D dose response curve for ATP ion channel expression density (x-axis) and glucose concentration (z-axis) against the sympathetic neurone action current frequency (y-axis) generated from several experiments such as those in **(C)**.

ATP-sensitive inwardly rectifying potassium (K_ATP_) channels were identified in spinally projecting neurones of the rat PVN by Li et al. (2010), where they mediate adenosine inhibition. K_ATP_ channels generally act as energy sensors (Nichols, 2006) and are closed by intracellular ATP. The first and most widely established role of K_ATP_ channels [beyond the regulation of insulin secretion in pancreatic β-cells (Ashcroft and Rorsman, 1990)] is to suppress electrical activity as ATP levels fall, and this has been shown in cardiac, skeletal, and smooth muscle (Wellman et al., 1999). In neurones, their role is central to glucose sensing; as glucose levels increase, so does intracellular ATP and this closes K_ATP_ channels and increases firing rate (Levin et al., 2004). Generally it is accepted that whilst hypothalamic neurones, including those in the PVN (Melnick et al., 2011), can be both excited or inhibited by glucose, only those excited by glucose are thought to involve K_ATP_ channels. Since sympathetic *activation* by hypoglycaemia (Fagius, 2003) is poorly understood, but an important part of autonomic glucose counter regulation (Evans et al., 2003), we investigated whether PVN K_ATP_ channels could also be involved with cellular inhibition of neuronal activity. Using a combination of mathematical modeling and patch-clamp electrophysiology (like that described above), we extended our sympathetic control neurone model to include K_ATP_ as the only “glucose” sensor. In our model, KATP is expressed in both spinally projecting neurones and their GABA-ergic input neurones. We found that this model is sufficient to account for *inhibition*, *no effect* or *activation*, simply depending on the balance of K_ATP_ channel conductance in the spinally projecting neurones themselves and the GABA-ergic inputs (**Figure 5**).

### Two-Pore Potassium Channels

Two-pore domain potassium (K2P) channels are a structurally distinct subset of the mammalian K^+^ channel superfamily widely distributed in the CNS, including the hypothalamus (Maingret et al., 2000; Hervieu et al., 2001; Medhurst et al., 2001; Han et al., 2003; Doroshenko and Renaud, 2009; Enyedi and Czirjak, 2010). The functional channel is a dimer with each subunit containing two pore-forming loops and four transmembrane domains (4TM/2P). In humans, 15 K2P channel genes (KCNK) have been identified, which can be divided into six distinct subfamilies on the basis of both their structural/functional properties; TWIK, TASK, TREK, THIK, TALK, and TRESK. One of these channels has been specifically implicated in PVN function; it was shown that PVN neurones have a background (leak) pH-sensitive TASK-like current with patch-clamp electrophysiology (Doroshenko and Renaud, 2009). This was manifested as a large shift in resting membrane potential in response to extracellular pH changes. This channel may be involved with appetite control, since the authors showed that orexin-induced currents from PVN neurones were mediated by suppressing its activity (Doroshenko and Renaud, 2009).

## Sodium Channels

Sodium channels are generally classified according to the trigger that opens them, for example, a voltage-change (termed “Voltage-gated” or “Na_v_ channel”) or binding of ligand (termed ligand-gated sodium channels). Sodium channels are important for generating electrical signals in neurons. Na_v_ channels are ubiquitous in the central nervous system (Yu and Catterall, 2003); their primary function in neurones is to generate and propagate action potentials (Catterall et al., 2005a). Sodium channels are of importance to the history of physiology; the work of Hodgkin and Huxley on sodium channels was a major milestone in electrophysiology (Hodgkin and Huxley, 1952).

In the brain, sodium channel proteins are composed of a complex of a 260 kDa α subunit in association with one or more auxiliary β subunits (β1, β2, and/or β3) of 33–36 kDa (Catterall, 2000a). Nine α subunits (Na_v_1.1–Na_v_1.9) have been functionally characterized, and a tenth related isoform may also function as a Na^+^ channel. The β-subunits are thought to be involved with trafficking or modulation of the α-subunit (Molinarolo et al., 2018), although exactly how is not entirely clear. The α-subunits, termed Na_v_1, are encoded by a family of genes called *SCNA*, of which, several have been discovered in the human genome. In total, 9 subtypes of Na_v_ have been identified in mammals (Na_v_1.1–1.9), with four α subunits found in the central nervous system; types I, II, III, and IV. In the CNS and heart, Na^+^ channels contain a mixture of β1–β4 subunits whereas in skeletal muscle, Na^+^ channels only contain the β1 subunit (Black and Waxman, 2013).

Tetrodotoxin, the guanidinium-containing blocker extracted from the Puffer fish, has contributed the most to our understanding of Na^+^ channels. Na_v_ channels can be classified by their sensitivity to TTX; SCN1A (Na_v_1.1), SCN2A (Na_v_1.2), SCN3A (Na_v_1.3), SCN4A (Na_v_1.4), SCN6A (Na_v_1.6), and SCN7A (Na_v_1.7) are blocked by low nanomolar concentrations of TTX; therefore, these subtypes are classified as TTX-sensitive, whereas, SCN5A (Na_v_1.5), SCN8A (Na_v_1.8), and SCN2A (Na_v_1.9) are inhibited by only high micromolar TTX concentrations and are considered TTX-resistant channels.

Most neurones express a high density of TTX-sensitive Na^+^ channels, carrying a large rapidly activating and rapidly inactivating ‘transient’ sodium current when membrane voltage is depolarized above threshold (typically near -55 to -50 mV). This current has rapid kinetics, reaching its peak in less than a millisecond and declining to baseline within a few milliseconds, mediating the upstroke of the neuronal action potential. However, in addition, there is also a small slowly inactivating or non-inactivating sodium current, constituting a steady-state persistent sodium current (INaP) (Stafstrom, 2007; Carter et al., 2012). So whilst there are several ion channel types that conduct sodium ions, this section will focus on the voltage-gated sodium channels (Na_v_) and epithelial sodium channels (ENaC) which have both been shown to be present in the PVN (**Table 4**).

**Table 4:** Other channels in the PVN.

Ion channel family	General role	Subtype Identified	Method	Relevance	Reference
**Sodium channels**					
Na_V_	Generate and propagate action potentials	Type I, type II, and type III	Immunohistochemistry, RT-PCR	Expression profiles:	Westenbroek et al., 1989; Furuyama et al., 1993; Black et al., 1994; Whitaker et al., 2001; Jarnot and Corbett, 2006
Voltage-activated				Type I weak	
				Type II and III strong	
ENaC Epithelial sodium channel	Sodium homeostasis	–	*In situ* hybridization	Weak immunoreactivity	Wang et al., 2010; Teruyama et al., 2012
**Calcium channels**		
Ca_V_	Involved in muscle contraction and excitation of neurones	L-type (Ca_V_1.x)	Immunohistochemistry	–	Chin et al., 1992; Hetzenauer et al., 2006
Voltage-activated		T-type (Ca_V_3.x) (Ca_V_3.1)	Patch clamp electrophysiology, RT-PCR, immunohistochemistry	Activation leads to depolarization, regulates action potential generation, bursting behavior and pacemaker activity Generate low-threshold spike.	Tasker and Dudek, 1991; Perez-Reyes, 1999, 2003; Luther and Tasker, 2000; Stern, 2001; Luther et al., 2002; Sonner and Stern, 2007; Lee et al., 2008
**Others**					
P2X-purinoreceptors	Mediate fast synaptic transmission	P2X1-6	Immunohistochemistry	These receptor channels are often linked to inflammation, but this has not been studied in the PVN (in relation to P2X)	Cham et al., 2006
ATP-gated					
ASICs	pH sensing	ASIC3	RT-PCR, Western blot		Meng et al., 2009
Acid-sensing					
HCN Hyperpolarization-activated, cyclic-nucleotide-gated	Modulating pacemaker activity	HCN1-4	RT-PCR	Hyperpolarization activated current responsible for pacemaker activity	Monteggia et al., 2000; Shirasaka et al., 2007


### Voltage-Gated Sodium Channels

Several studies have described the expression pattern of Na_v_ in the rodent CNS; however, these studies have focussed on expression rather than function (Westenbroek et al., 1989; Black et al., 1994; Whitaker et al., 2001). Using mRNA expression profiling the Na_v_ subunits I, II, III have been identified throughout the rat hypothalamus, including in the PVN. The spatial distribution patterns of mRNA for subunits I, II, and III were shown to be very different, with type I expression being weak, and types II and III much stronger (Furuyama et al., 1993; Black et al., 1994). In summary, a range of voltage-gated sodium channels are expressed in the PVN, but there is not enough information to assess what specific functions these different isoforms may confer.

### The Epithelial Sodium Channel (ENaC)

The epithelial sodium channel (ENaC) consists of combinations of four subunits: α, δ, β, and γ. These subunits are encoded by four genes: SCNN1a, SCNN1B, SCNN1G, and SCNN1D (Garty and Palmer, 1997). ENaC is best known for its role in Na^+^ reabsorption in the nephron, but the presence of ENaC in the PVN has been identified using *in situ* hybridization. Weak immunoreactivity for ENaC has been shown in the parvocellular region of the PVN and not in the magnocellular region (Wang et al., 2010; Teruyama et al., 2012). Teruyama et al. (2012) have shown that there is immunoreactivity for the α-ENaC subunit in both the parvocellular and magnocellular areas of the PVN. This immunoreactivity is co-localized with vasopressin and oxytocin only in the magnocellular cells of the PVN, suggesting a role in fluid regulation/diuresis within these cells. This group also performed voltage-clamp electrophysiology on magnocellular PVN neurones and applied the ENaC blocker benzamil, which reversibly reduced a steady-state inward current and decreased cell membrane conductance. Additionally, benzamil caused membrane hyperpolarization in the majority of vasopressin synthesizing magnocellular neurones and in around 50% of the oxytocin synthesizing neurones. ENaC in magnocellular neurones of the PVN will therefore affect firing patterns, and this in turn will ultimately control the secretion of vasopressin and oxytocin and fluid retention (Teruyama et al., 2012). Circumstantial evidence also suggests that PVN ENaC may contribute to hypertension. ENaC activity modulates sympathetic output and modulates blood pressure, an effect modulated by angiotensin receptors (Busst, 2013) known to be expressed in the PVN (specifically in spinally projecting autonomic neurones) (Oldfield et al., 2001). Furthermore, the salt-sensitive hypertension model (Liddle Syndrome) centers on the ENaC dependent elevation of cerebrospinal fluid sodium levels (Van Huysse et al., 2012).

## Voltage-Gated Calcium Channels

Encoded by the CACNA gene, calcium channels show high selective permeability to calcium ions. Often referred to as voltage-dependent calcium channels (VDCCs), as distinct from ligand-gated calcium channels. VDCCs are activated by depolarized membrane potentials and allow the flow of Ca^2+^ into the cell, which, depending on the cell type can result in a range of physiological processes such as excitation, gene expression or release of hormones/neurotransmitters. VDCCs are formed as a complex of different subunits: α_1_, α_2_δ, β_1-4_, and γ. The Ca^2+^-selective pore is formed from the α_1_ subunit which contains the voltage-sensing domain and drug binding sites (Catterall, 2000b) and associated subunits have several functions including modulation of channel gating. VDCCs can be divided into three categories depending on the subunits expressed: (1) High-voltage activated dihydropyridine-sensitive L-type CACNA1 (Ca_v_1.x) channels, (2) High-voltage activated dihydropyridine-insensitive CACNA1 (Ca_v_2.x) channels and (3) Low-voltage activated T-type CACNA1 (Ca_v_3.x) channels, all expressing a variety of subunits (Catterall et al., 2005b).

L-type CACNA1 (Ca_v_) channels have been identified in both magnocellular and parvocellular regions of the rat PVN using *in situ* hybridization and immunohistochemistry (Chin et al., 1992) (**Table 4**). Hetzenauer et al. (2006) investigated the presence of the dihydropyridine-sensitive L-type calcium channels CACNA1C (Ca_v_1.2) and CACNA1D (Ca_v_1.3) throughout the mouse brain (Hetzenauer et al., 2006). By using the dihydropyridine-sensitive L-type calcium channel activator BayK 8644 in the mutant Ca_v_1.2^DHP-/-^ mouse, it was possible to determine whether the increase in neuronal activity (using *c-fos* as an indicator) was due to CACNA1C (Ca_v_1.2) or CACNA1D (Ca_v_1.3). Within the PVN, *c-fos* in BayK treated mutant mice was markedly increased compared to other areas of the brain, suggesting this area is predominantly CACNA1D (Ca_v_1.3) L-type calcium-mediated activation, rather than CACNA1C (Ca_v_1.2) (Hetzenauer et al., 2006). Although L-type VDCCs are present throughout the PVN, the predominant VDCC current identified in parvocellular cells to date is the (transient) T-type Ca^2+^ current (as opposed to voltage-gated K^+^ channels in magnocellular cells of the PVN as discussed earlier) (Tasker and Dudek, 1991; Luther and Tasker, 2000). T-type Ca^2+^ channels modulate neuronal function via regulation of Ca^2+^ influx into the neurone, leading to cell depolarization and action potential firing. Specifically, these channels are important in generating and regulating action potentials, influencing pacemaker activity and action potential bursting behavior (Perez-Reyes, 1999, 2003). Patch-clamp recordings made from retrogradely labeled neurosecretory and non-labeled non-secretory parvocellular cells of rat PVN, showed that non-secretory parvocellular cells generate a low-threshold spike via T-type Ca^2+^ channels, whereas this is not seen in identified neurosecretory neurones (Stern, 2001; Luther et al., 2002; Sonner and Stern, 2007). This study further confirmed the idea that different groups of cells in the parvocellular area of the PVN exhibit different membrane properties. Additionally, more recent studies involving a combination of RT-PCR, immunohistochemistry and patch-clamp recordings have indicated that CACNA1G (Ca_v_3.1) is the major subtype of channel expressed in pre-autonomic cells mediating the T-type Ca^2+^ dependent low threshold spikes (Lee et al., 2008). These results provide clear evidence that modulation of these channels is vital for normal activity of pre-autonomic PVN neurones.

## Other Ion Channels

Above we have discussed the major families of ion channels identified in the PVN and finally we cover ion channels where there are only isolated studies of expression in the PVN (**Table 4**). For example, the P2 purinergic membrane receptor family comprises several subtypes that are commonly activated by extracellular nucleotides (ATP or ADP). P2X receptor channels are ligand-gated ion channel receptors (P2X1-7), whereas P2Y are G-protein coupled receptors. Double-labeling fluorescence immunohistochemistry shows that, in the PVN, vasopressin-containing neurones largely also expressed P2X4, P2X5, and P2X6 receptors, while oxytocin-containing neurones expressed P2X4 receptors. Since purinergic pharmacology is relatively advanced compared to many of the other channels discussed above, these constitute ideal future candidates for experimental analysis of PVN regulation of autonomic control (Guo et al., 2009).

Acid-sensing ion channels (ASICs) are proton-gated voltage-insensitive cation channels, responsible, as their names suggests, for sensing pH. In mammals, ASIC are encoded by five genes that produce ASIC protein subunits: ASIC1, ASIC2, ASIC3, ASIC4, and ASIC5. Immunoreactivity has been shown to be high in the rat PVN for ASIC3 (Meng et al., 2009) and ASIC4 mRNA has been identified within the PVN using radioactive *in situ* hybridization (Hoshikawa et al., 2017), suggesting an acid sensing role for neurones within this area. However, no functional studies have been performed.

Pacemaker activity of some PVN hypothalamic neurones is modulated by a hyperpolarization activated current (I*h*) (Shirasaka et al., 2007). A family of channels, hyperpolarization-activated, cyclic nucleotide-gated (HCN) channels, has been identified as responsible for this pacemaker activity in neuronal cells. A study by Monteggia et al. (2000) identified four members of this family HCN1-4 with verifying expression patterns throughout the rat brain. All members of this family were shown to be present within the PVN; with expression profiles for mRNA ranging from weak for HCN2 to extremely strong for HCN3 (Monteggia et al., 2000).

Surprisingly, although dogma states that anion channels, such as chloride channels, would be present in the plasma membrane of all cells, no studies have been reported on their presence in the PVN. Chloride channels are functionally and structurally diverse with a range of functions such as cell volume regulation, regulation of pH and cell membrane potential control. As one of the main functions of these channels is to regulate excitability of neurones it is likely that chloride channels would be present within the PVN (Hille, 1986).

A vast variety of ion channels exist in mammalian biology, each with diverse physiological functions. It is likely that each channel subtype will contribute to the resting membrane potential of PVN neurones with the ability to modulate firing activity and the more we know about these the better our predictive neuronal models will be and the closer we will be to selectively modulating particular neurones without adversely effecting others. There will be many additional PVN channels that are not discussed here, some of these have been include in the tables, and others are, naturally, yet to be discovered.

## PVN Ion Channels in Disease

The PVN is linked to several important physiological processes and one of the exciting prospects for the future is to uncover whether changes in PVN ion channel expression or activity might contribute to pathological changes and could be pharmacologically (or biologically) interdicted. This is because there are several ion channel modulating drugs on the market and many new ones in production (Humphries and Dart, 2015). To date, there are a few lines of direct evidence that PVN ion channel changes underlie diseases; such evidence principally focusses around four conditions; diabetes, hypertension, heart failure and hormonal changes associated with depression.

### Diabetes

In a mouse model of spontaneous type 2 diabetes (db/db), it was shown that identified liver-related PVN neurones were significantly more active (the majority fired spontaneously) compared to those of control (lean) mice (where the majority of neurones were silent) (Gao et al., 2017). This could be explained by an increase in TRPC channels, since mRNA for TRPC1,4,6 and 6 were all show, by qPCR to increase in the streptozotocin T2D model (Zheng et al., 2014).

### Hypertension

Two separate lines of evidence implicate ion channel changes in hypertension. We described above that the small Ca^2+^ sensitive potassium channel (SK) expression was decreased in spinally projecting (sympathetic) neurones in hypertension (Pachuau et al., 2014). This could either be contributory to hypertension or resultant from hypertension, but since decrease in expression would be expected to increase neuronal activity the former seems more likely. Over expression of glutamate receptors also contributes to sympathetic hyperexcitability in spontaneously hypertensive rats (Li et al., 2014), although this type of receptor plasticity could be secondary to elevated angiotensin levels, since Glass et al. (2015) showed that NMDA levels increase in neuronal nitric oxide synthase (nNOS)-expressing PVN neurones following just 2 weeks of angiotensin treatment leaving animals with elevated blood pressure. Together these data suggest that PVN NMDA receptor plasticity may well contribute to development of hypertension.

### Heart Failure

The PVN has been linked to cardiovascular diseases such as hypertension and heart failure, where an elevation in sympathetic nerve activity is observed (Zhang et al., 2002; Guyenet, 2006). For example, dysfunction of the tonic regulation of the PVN has been suggested to play a role in progression of heart failure (Pyner, 2014). In addition, lesioning of the PVN prevents the development of spontaneous hypertension in SHRs (Takeda et al., 1991). Interestingly, PCR data showed that mRNA expression levels of α1 subunit and β1 receptors were significantly lower in a rat model of chronic heart failure, suggesting that tonic inhibition (mediated by GABA receptors in the PVN) of sympathetic output may be blunted in chronic heart failure (Wang et al., 2009).

Over expression of kainate receptor-channels also contributes to sympathetic hyperexcitability in spontaneously hypertensive rats (Li et al., 2014). Additionally, inhibited GABA-ergic neurotransmission contributes to this phenomenon in models of heart failure (Patel, 1997; Pyner, 2014). Briefly, sympathetic output is elevated in the chronic heart failure model, at the level of the PVN (Zhang et al., 2002; Li and Patel, 2003), following changes in reactive oxygen/nitric oxide pathways together with increases in pro-inflammatory cytokines and prostaglandins (Zubcevic et al., 2011). Plasma cytokines are elevated in male mild hypertensives (Chae et al., 2001) and this changed neuroinflammatory status of cardiovascular control nuclei appears to be of central, rather than systemic origin (Waki et al., 2008). It is not clear whether this is causal or resultant from hypertension, but it seems an important phenomenon. Rats with induced heart failure have reduced nNOS (Zhang et al., 1997, 2002). Additionally, cytokines in the PVN up-regulate AT-1R expression and deplete nNOS (Guggilam et al., 2008). Since nNOS produces NO that facilitates tonic inhibition of PVN GABA neurotransmission, this dis-inhibition scheme serves to exaggerate sympathetic output in heart failure rats. Ultimately, block of cytokines (TNF in particular) appears to limit the overall PVN sympathoexcitation (Guggilam et al., 2007, 2008, 2011).

### Other Diseases

Hypertension and heart failure are not the only diseases mediated by GABA neurotransmission, and since the PVN is at the core of autonomic-endocrine integration, it is no surprise that it is involved in a variety of diseases/disorders. For example, HPA stress responses are initiated by CRH neurones within the PVN and the HPA axis are known to be activated in depressive disorders (Varghese and Brown, 2001). Remarkably, the total number of CRH-immunoreactive neurones is four times higher in depressed patients compared to healthy individuals (Raadsheer et al., 1993) despite an overall reduction in total PVN neurone numbers (50%) in major depression and bipolar patients (Manaye et al., 2005). In a recent study, Gao et al. (2013) looked at both isoforms of glutamic acid decarboxylase (GAD_65/67_) (the synthetic enzyme for GABA) as a marker to monitor GABAergic neurotransmission in the PVN. They reported a reduction in the density of GAD_65/67_ in the PVN of major depressive disorder patients (Gao et al., 2013). They also reported a negative correlation between the density of GAD_65/67_-immunoreactive (ir) and the number of CRH-ir neurones in the PVN in the depression group, but not in the control group. Interestingly, no change in neuronal numbers was observed in other areas of the hypothalamus such as the supraoptic nucleus (Gao et al., 2013).

## Limitations

In this review, we provide a summary of our knowledge of PVN ion channel expression and function to date. In the above passages we address all ion channel studies we are aware of that was conducted in the PVN. It should be noted that each type of study has its strengths and weaknesses, but we take each authors *peer reviewed* data at face value, neutrally and without judgment. For example, the physiological and electrophysiological approach we adopt ourselves is heavily dependent upon ion channel pharmacology, it has the strength that you can see what the channel does or determine its electrical fingerprint, but the agonists and antagonists are rarely as selective as one would wish. There are molecular approaches available, siRNA for example, but these are very difficult to deploy in native tissue. Whilst it would be considered controversial for some, even ordinary knock-down studies have the profound limitation that we know considerable compensatory expression will take place and muddy conclusions drawn. qPCR, microarray or even RNA sequencing are powerful approaches, but unless backed-up with some other type of study, show only the transcript expression not the ion channel protein itself. Even if backed up by Western blot analysis, these studies do not reveal whether there is patent membrane expression or whether ion channel function is compromised or altered. Immunohistochemistry can often suffer from issues of non-selective binding etc., unless properly validated, but again it does not tell us about the functional integrity of the channel. It is also very difficult to definitively link, directly, any of the protein or mRNA data directly to the patch-clamp data presented, or visa-versa.

## Conclusion

In this review we have brought together information on the expression and role of a wide range of ion channels in the PVN. Since the PVN is central to autonomic and endocrine regulation of homeostasis these channels may in the future emerge as therapeutic targets. Our own research focuses on cardiovascular control, but there is clear potential for intervention in a wide range of areas. Several challenges remain; least of all not access to this area of the forebrain. Clearly the future will lead not just information on which ion channels are present and potential targets for intervention, but mechanisms to get that drug or biologic to the target area. In the work of Geraldes et al. (2014), this was achieved by intracranial microinjection and our own unpublished work shows we can deliver biologics to the PVN via spinal injection, but these are still not attractive avenues for medical deployment of treatments. Of course, most centrally acting drugs; sedatives, anesthetics and supraspinal analgesics are effective without any such targeting. This may too prove useful for therapeutic targeting of PVN ion channels. The diversity of ion channels, receptors and neurotransmitters within the PVN (Pyner, 2009; Nunn et al., 2011) is such that there may be targets that can be preferentially and beneficially activated with systemic application, as for other neuropharmacological agents, albeit they have largely been discovered by serendipity rather than design. Another challenge will be continuing the identification of biophysical and pharmacological profiles of specific subtypes of PVN neurone. Whilst the PVN is a relatively small nucleus comprising about 1% of the brain, data from different studies are not easily separated into different categories of neurone. Our work has modeled the biophysical properties of spinally projecting or parvocellular neurones, but these will likely consist of mixed populations. The earliest studies identified the so called type I and II neurones and this advanced recently with the single cell PCR work of Lee et al. (2012). Since it is believed that type II neurones largely constitute pre-autonomic neurones, this allows for refinement of predictive models of neuronal behavior. However, we are far off understanding the PVN as a network, with differential outputs to cardiovascular and other targets and inputs from the several sources the PVN receives. The rapid accumulation of ion channel data over the past 20 years does, however, already give considerable optimism for rationally designed therapeutic interventions in the future.

## Author Contributions

All authors listed have made a substantial, direct and intellectual contribution to the work, and approved it for publication.

## Conflict of Interest Statement

The authors declare that the research was conducted in the absence of any commercial or financial relationships that could be construed as a potential conflict of interest.
